# General Certificate of Secondary Education (GCSE) and the assessment of science practical work: an historical review of assessment policy

**DOI:** 10.1002/curj.20

**Published:** 2020-01-10

**Authors:** Ann Childs, Jo‐Anne Baird

**Affiliations:** ^1^ University of Oxford UK

**Keywords:** science practical work, curriculum and assessment policy, high‐stakes testing

## Abstract

This article responds to two key concerns in science education: firstly, that policies designed to assess practical work have distorted its use as an effective pedagogical tool. Secondly, it addresses concerns about the lack of research on the assessment of practical work. The article analyses the policy trajectory for the assessment of science practical work, through the GCSE, in the English National Curriculum from 1988 to the present day. Drawing on published research and policy documents, using Bowe, Ball and Gould's policy cycle approach to policy analysis, we first identify three distinct eras which represent different ways of assessing practical work from 1988 to the present day. Second, we demonstrate that the interaction between high‐stakes assessment narrows the ways practical work is conducted in schools. The interaction between curriculum policy and assessment policy and its influences on pedagogy for practical work has been influenced by the high‐stakes nature of the tests. This is not a unique case to England nor to science education. Finally, we question whether we can design assessments, ‘tests worth teaching to’, that can withstand the implications of high‐stakes testing.

## Introduction

Science education is widely recognised as not only an important part of the compulsory curriculum, but as a foundation for life skills and an underpinning of industrial and economic policy. Debates about the nature of science that should be taught in schools are perennial, including the balance between skills and knowledge (Matthews, 1994/2014; Erduran & Dagher, 2014; European Commission, 2015). In these debates the role and importance of practical work as a key teaching and learning strategy in science classrooms in England and internationally has also been argued and debated over for many years (see e.g. Buchan & Jenkins, 1992; Gee & Clackson, 1992; Donnelly, 1994; Jenkins, 1995b; Roberts & Gott, 2006; Abrahams & Millar, 2008). More recent work in England has questioned both the effectiveness of practical work as a teaching and learning strategy and its ability to motivate students to learn science (see e.g. Abrahams & Millar, 2008; Abrahams, 2009). In response to these concerns, particularly that practical work has a focus on ‘doing’ rather than on conceptual development, Abrahams (2010) argued for a more ‘minds on approach’ to practical work. Amongst these debates concerns have also been expressed about the assessment of practical work, particularly that high‐stakes assessments^1^ have distorted the ways practical work has been used to facilitate effective teaching and learning in science classrooms (see e.g. Keiler & Woolnough, 2002; Abrahams *et al*., 2013; Wilson *et al*., 2016). The assessment of practical work has taken place in England for many years. Indeed, Buchan and Jenkins (1992) reported that practical assessment of practical work in three‐hour examinations was taking place at A level as early as 1963. However, Abrahams *et al. *(2013) have argued that little research has focused on this area. Given the concerns expressed about the ways practical assessment has distorted the use of practical work as a teaching and learning strategy, along with the lack of research on practical assessment over time, this article takes a specific case, the policy trajectory for the assessment of science practical work in General Certificate of Secondary Education (GCSE) examinations in England. GCSEs were introduced in 1986 to reflect the comprehensive schooling system to assess the entire ability range. In addition, coursework and practical work of various kinds were introduced into the GCSE examinations at their inception in an attempt to assess skills that went beyond the strictures of what could be assessed in an examination hall (Secondary Examinations Council, 1985, 1986). GCSE has been chosen as the case because virtually all pupils in England sit these examinations and they are high‐stakes assessments. High‐stakes testing is defined as an assessment which has important consequences (e.g. promotion, salary, job retention) for students, teachers, administrators or schools. In the case of England, GCSE results are used as measures of school quality in government inspections and also in constructing league tables of secondary schools' examination results for parents. This case will be used to examine curriculum and assessment policy, illustrating (a) how views of the underlying assessment construct and curriculum change over time, (b) how these are influenced by different actors, (c) the effects of a high‐stakes assessment context for pupils and teachers and (d) how broader assessment policy matters affect specific subjects. In illustrating these points, we seek to add to the literature in the assessment of practical work in science but also to investigate in depth the ways in which curriculum and assessment policy in science interact to influence how practical work is used to support learning in science.

Pedagogical purposes and educational principles behind the learning, curriculum and assessment of GCSE practical science are demonstrably influenced by the non‐pedagogical. However rather than viewing practical science assessment policy as an externally imposed, ongoing game of performativity (Priestley & Philippou, 2018), we argue that it is not only accountability measures that are driving a wedge between curriculum and assessment. We develop this argument in the discussion and conclusion where we also show that this case has lessons for curriculum policy more broadly.

## Theoretical framework

Our theoretical framework to undertake this policy analysis is the policy cycle outlined by Bowe *et al. *(1992). Bowe *et al*.'s policy cycle approach challenges the separation of policy, politics and practice (White & Crump, 1993, p. 415) by drawing attention to the work of policy recontextualisation that occurs in schools as a policy is implemented. In addition, the policy cycle allows for an analysis of how interested parties, in this case science education academics, teachers, examination boards and policy makers, interact and interpret policy. Three primary policy contexts were identified in the policy cycle: the first is the context of influence ‘where public policy is normally initiated’ (p. 19). The second is the context of policy text production in which policy texts are produced that *represent* policy. Bowe *et al. *(1992) explain that these representations can take the form of official legal texts and policy documents such as the Education Reform Act and National Curriculum documents referred to later in this study. They can also be politicians' or officials' speeches or they can be ‘formally and informally produced commentaries’ whose purpose is to make sense of official texts (p. 21) such as the materials produced by examination boards to support teachers in conducting and assessing science practical work. The final context is the context of practice, where policy is interpreted and constructed. In this way, policy can be:

thought of as texts constituted by discourses. Policy is thus seen as a representation which is encoded and decoded in complex ways. Policy texts may be ‘readerly’ or ‘writerly’, according to the degree of interpretation allowed to the reader, but always and inevitably texts are interpreted and thus contested, adopted and adapted in different contexts of work. Thus, policy is constantly being made or remade at different educational sites. (Bowe *et al*., 1992, p. 22)

More readerly policy texts are characterised as being more prescriptive, for there to be a ‘minimum of creative interpretation by the reader’ and provide the National Curriculum (NC) texts produced in 1988 with ‘technical language, attainment targets, standardised testing and programmes of study’ as one example (Bowe *et al.*, 1992, p. 11). In contrast, more writerly policy texts are characterised as being more open to interpretation and allow for a more critical and creative’ response to the text (Bowe *et al.*, 1992, p. 11). This characterisation comes from Bowe *et al*.'s reading of the work of Roland Barthes' work as discussed in Hawkes (1977):

Literature may be divided in to that which gives the reader a role, a function, a contribution to make, and that which readers the reader idle or redundant, ‘left with no more than the poor freedom to act of reject the text’ (S/Z: 4) and which thereby reduces him to that apt but impotent symbol of the bourgeois world, an inert consumer to the authors' role as producer. (113–114)

Other researchers in educational policy have adopted this characterisation (see e.g. Conner & James, 1996; Hall, 2001) as a useful tool for the analysis of policy text but others have criticised it. For example, Hatcher and Troyna (1994) have questioned how appropriate classifying policy texts as readerly or writerly based on a characterisation intended for literary texts is when they have different functions:

Unlike texts, policies have to be put into action in the real life of institutions in order to ‘work’. Authors cannot *impose* a response at the level of discourse, whatever their ‘readerly’ or ‘writerly’ intentions. In contrast, the state, while it cannot also impose an interpretation at the level of discourse, certainly can impose one at the level of practice, and its ability to do so operates at the level of policy, not discourse. (p. 163)

However, despite their contention that ‘Barthes notion of readerly and writerly texts obscures more than it illuminates’ they do concede that:

the Standard Assessment Tasks (SATs) associated with the National Curriculum and the recent orders governing the dissolution of the binary system in Higher Education in Britain come close to this notion of a ‘readerly’ policy text. (p. 163)

Broader critiques of the policy cycle essentially argued that there was too much emphasis upon the agency of teachers and schools (Lingard, 1993; Hill, 2001) and a lack of emphasis upon the state (Hill, 2001) or supranational effects (Lingard, 1993). In our use of the framework, we consider the interaction between various actors, including teachers, the state, examination boards and the international literature on science education. Although there clearly are policy effects of supranational trends and actors upon assessment policy, they are at a broader level and therefore not the subject of this article.

Broad societal and educational changes have impinged upon the assessment of practical science in GCSEs, as well as more proximal factors regarding the politics and policy of assessment and debates related to the nature of science education. The article begins with a short consideration of the reasons for the introduction of the NC in England and Wales (context of influence). We then focus on the science NC and look first at the role of the Science Working Group (SWG) who produced the 1989 Order including the introduction of science practical assessment, centred on science investigation (context of influence). Next, we narrow the discussion to the different manifestations of practical assessment for the Science GCSE (context of text production), which have been mandated and implemented (context of practice) in the period 1989 to the present day. The manifestations of practical assessment at GCSE, as described above, can be divided into three policy eras (Table 1). Whilst valuable more generally, we do not use Hodgson and Spours' (2006) notion of an ‘education state’ between the policy contexts and the eras because the examination boards and their relations with the wider education system are not the focus of the article.

**Table 1:** GCSE practical science assessment policy periods

Era	Years	Performance conditions	Marking and quality control	Weighting on examination outcome (%)
1. Coursework assessment through practical investigations	1992–2006	Conducted throughout the course; in the classroom, as homework or a mixture of both	Assessed by teachers and moderated by examination boards	25
2. Coursework assessment through controlled assessments	2006–2016	Conducted in the classroom under supervision	Assessed by teachers and moderated by examination boards	25–40
3. Written examination	2016	Examination conditions	Examiners and sampled by supervisors	Minimum 15

## Approach to assessment policy analysis

We present a narrative, critical evaluation, utilising published research and policy documents. This method was selected instead of a systematic review because our method involved utilising the policy framework to analyse the key developments rather than primarily to investigate previous publications per se. A systematic search for relevant publications was conducted using Google scholar, Google search engine and ERIC (Education Resources Information Center, a database of literature related to education), using the following search keywords: Sc1^2^ assessment, practical work assessment, National Curriculum policy, science National Curriculum, controlled assessment. This resulted in a core of 48 academic articles, policy papers, government reports, examination board reports on practical assessment and examination board guidance being identified using the following inclusion principles:

- Articles prior to the time frame which allow for an understanding of the historical role of practical work and practical investigations in the English context;
- Articles being within the time frame of the policy analysis 1988 to the present day;
- Relevant to the assessment of GCSE coursework in England and Wales (thus including one article about assessment of Sc1 assessment at Key Stage 4).

The following procedures for analysis were employed:

1. Each document was classified into the era in which it was particularly relevant and some fell into more than one era.
2. The documents from each era were read and, where relevant, were matched to the three contexts of influence outlined from the Bowe *et al. *(1992) policy framework and this was recorded in a 3 × 3 matrix (see Table 2).
3. Finally, papers in each cell of the matrix were read and for each context key questions were used to interrogate the literature and these questions can be found in Table 2.

**Table 2:** Documents for policy analysis

Era	Context of influence	Context of text production	Context of practice
	Which key government, national bodies, representative groups, for example, influenced the development of the NC and the science NC and practical work assessment policy?	What policy texts were produced in the particular era and what did they contain?	How were the policy texts interpreted in practice and what issues and challenges occurred as policy was implemented?
	Which key actors, if any, were involved in influencing the development of the NC and the science NC and practical work assessment policy?	In what ways and to what extent did these texts allow the reader freedom to interpret them, i.e. to what extent were they more readerly or writerly?	
1. Coursework assessment through practical investigations 1992–2006	Black (1993)	Black (1995a)	Bishop *et al. *(2006)
	Black (1995a, 1995b)	Donnelly *et al. *(1996)	Black (1995a, 1995b)
	Bryce and Robertson (1985)	Jenkins (1995a)	Donnelly (1994)
	Buchan and Jenkins (1992)	Jenkins (2009)	Donnelly (2000)
	Daugherty (1995)	National Curriculum Council (1991)	Donnelly (2001)
	Donnelly (1994)		Donnelly *et al. *(1996)
	Donnelly *et al. *(1996)		Hume and Coll (2008)
	ERA (1989)		Ipsos Mori (2006)
	Gee and Clackson (1992)		Jenkins (1995a)
	Hodson (1985)		Jenkins (2000)
	Jenkins (1995a, 1995b)		Jenkins (2009)
	Lawton (1994)		Nott and Wellington (1999)
	Roberts and Gott (2006)		House of Lords (2006)
	Secondary Schools Council (1985, 1986)		Millar (2011)
	Taylor (1995)		QCA (2005, 2006a, 2006b)
			Tytler and Swatton (1992)
2. Coursework assessment through controlled assessments 2006–2016	Coe (2007)	Edexcel GCSE Science Controlled assessment guidance for teachers	Score (2009)
	Millar and Osborne (1998)	OCR	Ofqual (2013)
	Ofqual (2013)	Twenty first century guide to controlled assessment	Ofqual (2015)
	Roberts and Gott (2006)	AQA Guide to controlled assessment	
3. Written examination 2016	Gove (2010)	AQA GCE chemistry: Required practical handbook	
	House of Commons (2012)	Clackson and Wright (1992)	
	Ofqual (2015)		

For each context themes were identified, for example, in Era 1 four key themes which constituted issues and challenges were identified in the context of practice which were: (a) Sc1 as a radical departure from previous teaching and assessment practices in science education; (b) the narrowing of science practical experiences in schools; (c) epistemological concerns about the nature of science investigations; and (d) assessment challenges. The themes emerging from each era and from each context are presented in the findings section below.

## Results

The results are presented for each of the eras shown in Table 1. For each era, the contexts of influence, policy text production and practice are discussed with the discussion shaped by the key questions in Table 2 and the themes identified.

### Era 1: 1992–2006—coursework assessment through practical investigations

#### Context of influence A—development of the National Curriculum

Various strands of right‐wing ideology present in the 1970s and 1980s led to the development of the NC in England and Wales. First, there were concerns about ‘the very diverse curriculum offerings in the upper years of secondary schools’ and corresponding lack of control over what was being taught (Black, 1995a, p. 160). Second, ‘a breed of emerging right wing leaders who had risen through the selective, state school system … were conscious of the loss of style of work they had enjoyed’ and were critical of the changes in the examinations and assessment which ‘were seen as part of a general threat to standards’ (Black, 1995a, p. 160). Concern for traditional models of education and society more broadly featured in this notion of slipping standards (Lawton, 1994, p. 144). However, Taylor (1995) also argues that it was not only the right wing of the political spectrum that was concerned with slipping standards. James Callaghan, the Labour Prime Minister, in his Ruskin College speech in October 1976 also argued for a vision of schooling that is ‘both rigorous and relevant’ (Taylor, 1995, p. 162). However, he goes on to acknowledge that, although Callaghan may have started the move towards an NC, it was Margaret Thatcher in 1998 who was responsible for what he perceived as ‘a highly prescriptive and contentious National Curriculum’ (Taylor, 1995, p. 163).

Finally, a further, right‐wing, ideological strand was a concern to marketise education, such that ‘schools should become independent and competing organisations, with primacy being given to parental choice of schools’ (Black, 1995a, p. 162) free from the bureaucratic control of local authorities. Bringing together the agenda of standards and parental choice, Donnelly *et al*. (1996) argued that in the development of the NC:

The government placed particular emphasis on assessment. Perhaps the central motivation for this emphasis was the view that assessment of outcomes would help constitute an educational market place in which parents would be the main players. (p. 5)

These neoliberal strands of market, competition, regulation of standards and market choice came together and led to the NC in 1988 (Black, 1995a, p. 162).

#### Context of influence B—development of the science NC and the introduction of science investigations as the form of practical assessment

The SWG, given the task of developing the science order of the NC, was chaired by Professor Jeff Thompson (University of Bath). Other members of the group consisted of primary and secondary teachers, academics, local education authority staff and an industrialist (Donnelly *et al.*, 1996, p. 7). According to Black (1995a, p. 166), this group was highly respected in the science education field in the UK. Historical factors regarding the role of practical work in science education in the English policy context were important at that point too: practical work as part of the science NC had already been long established in England (see e.g. Buchan & Jenkins, 1992; Gee & Clackson, 1992; Donnelly, 1994; Jenkins, 1995b) and could be traced back to the Great Exhibition of 1851 (Roberts & Gott, 2006, p. 45). Researchers in science education (see e.g. Buchan & Jenkins, 1992; Jenkins, 1995a) noted that scientific investigations had also been a part of the science educational landscape through initiatives such as the Nuffield Science Teaching Project (NSTP) which began in 1992 and the assessment of practical work by teachers was already taking place before the introduction of the NC in 1982 though not through science investigations (Bryce & Robertson, 1985).

As well as the contextual influences outlined above another key influence, significant in influencing the work of the SWG, was the Task Group on Assessment and Testing (TGAT), led by Professor Paul Black, set up by the then Conservative Minister of State for Education Kenneth Baker to advise on a new framework for national assessment. This reported on Christmas Eve 1987 and was published by the Government in January 1988 (Daugherty, 1995, p. 22). Donnelly *et al. *(1996) commented that elements of the proposals showed the influence of progressive educational thought (p. 5) particularly in promoting diagnostic and formative assessment and teachers' professional development and, at a technical level, the use of criterion referencing as the assessment paradigm (p. 5). However, as Donnelly *et al. *(1996) stated, a particularly ‘striking characteristic’ linked to criterion referencing was the creation of attainment targets and the recommendation that each attainment target had to be set out as criteria across ten levels, and testing in the form of standard assessment tasks (SATs) would take place at age 7, 11 and 14, with testing at age 16 being in the form of the GCSE (p. 5). However, the move to 10 levels of assessment is justified by Black (1993) as being a necessary part of the progressive move in the TGAT report to more formative assessment which, he argued, required ‘a criterion‐referenced approach’ (Black, 1993, p. 351).

In this scenario, therefore, the SWG were already beginning their work in the context of a significant legacy of the role and practice of practical work in England. Additionally, as will be shown, their work was to be constrained by the recommendations of TGAT. Commenting on these issues, Donnelly *et al. *(1996) wrote that one of the biggest controversies Sc1 faced from 1991 to 1994 ‘stemmed mainly from the problems of developing workable classroom practice and the demands of assessment’ (p. 9). Set out in the 1989 Education Reform Act, the SWG were asked to produce a structure for the science NC based on:

1. The knowledge, skills and understanding which pupils of different abilities and maturities are expected to have by the end of each key stage (in this Chapter referred to as ‘attainment targets’).
2. The matters, skills and processes which are required to be taught to pupils of different abilities and maturities during each key stage (in this Chapter referred to as ‘programmes of study’). (Education Reform Act, 1989)

#### Context of policy text production

In writing the science NC from the mandates handed down from the Education Reform Act and the TGAT report the SWG report treated ‘science as a single subject, with the weight of two normal subjects in the upper secondary years’ (Black, 1995a, p. 167) and initially proposed 22 attainment targets grouped into five profile components:

- Science content
- Practical investigation
- Communication
- The nature and history of science
- The applications and social implication of science

The final text implemented in 1989 was simplified, containing only 17 attainment targets based around three profile components. However, by 1990 it was realised that the practicality of assessing 17 attainment targets at 10 levels presented significant challenges (National Curriculum Council, 1991; Black, 1995a, p. 168). Therefore, a further process of revision was undertaken and the revised version in 1991 contained four attainment targets:

- AT1 (now called Sc1): Scientific investigation which incorporated some of the previous AT17 (Nature of Science);
- AT2: Life and living things;
- AT3: Materials and their properties;
- AT4: Physical processes.

Although some of AT17 (Nature of Science) was incorporated into AT1 (or Sc1), many argued that in fact much of this aspect was in essence discarded (see e.g. Jenkins, 2009). As will be seen later this will have implications for the type of practical investigative approach that was adopted as the means for assessment for practical work at GCSE.

The process of revision of the SWG's proposals from the publication of its report in 1988 to 1991 is a little unclear. Donnelly *et al. *(1996) initially perceived that the ‘Science Working Group (and TGAT), with published terms of reference, provided a degree of independence from the government’ (p. 217) and, at this stage the ‘science educationalists in the SWG were largely in control’ reflecting ‘privileged, or perhaps the marginal position of science within political and public life’ (p. 217). However, this position of control by educationalists on the SWG seems to have shifted when ‘other influences, beyond that of science educationalists came into play’ (p. 217). Efforts by ministers and civil servants to shift the balance of power away from TGAT and the SWG mean that later efforts to revise the orders resulting in the 1991 revision were done ‘in a less formally constituted, not to say private setting of the later groups’ (p. 217). Black (1995a) identifies one of these ‘later groups’ as being ‘a small group of national inspectors’, who were given six months to produce new proposals which reduced the 17 ATs published in 1989, as said above, to just 4 in 1991 (p. 168).

A third version of the science NC was published in 1995 in response to teacher strikes and protests about the excessive assessment burden on teachers (particularly AT1 [Sc1]: scientific investigation) from teacher assessment of Sc1. The group that produced this third version of the NC, according to Black (1995a), was ‘composed of almost entirely practising teachers’ who were given nine months on a part‐time basis to do so (p. 168).

It is Sc1 Experimental and Investigative Science around which assessment of practical work focused at GCSE and this is the focus of the first era, which lasted 14 years. In 2000 the NC was revised again and Sc1 now incorporated additional material under the heading ‘ideas and evidence’, and was renamed ‘Scientific Enquiry’.

The account above shows how the decision to move to an NC in science could suggest policy texts that, according Bowe *et al. *(1992), are characterised by a more readerly orientation because, as they claim:

an initial reading of National Curriculum texts, for example, and their technical language of levels, attainment targets, standardised attainment testing and programmes of study suggest such a readerliness. (p. 11)

However, evidence suggests that the intentions of the SWG for Sc1 and practical investigations was to make practical work more open, exploratory, creative and reflecting better how scientists work, giving a writerly tone (Jenkins, 1995a; Donnelly *et al.*, 1996). However, as will be seen in the next section, the implementation of Sc1 and a proliferation of a number of additional policy texts and guidance meant that in practice the overall policy text, the original NC document and the accompanying texts to support the implementation of Sc1 together became very prescriptive, with very readerly implications, leaving, as Bowe *et al. *(1992) would contend, ‘minimum opportunity for creative interpretation by the reader’ (p. 11).

#### Context of practice

This section outlines how the NC policy text for what became called Sc1 coursework assessment was taken into the arena of practice. We look specifically at the issues and challenges of practice that it created, leading ultimately to its being abandoned in 2006. As discussed above, the intentions of those responsible for the introduction of science investigations were to make practical work more creative and closer to how scientists work. The key reasons for the problematic implementation of Sc1 are presented in the following sections:

##### Sc1 as a radical departure from previous teaching and assessment practices in science education

The first key challenge for Sc1 was its assessment. Donnelly *et al. *(1996) argued that there seemed to be no firm basis of a shared understanding within the science education community, including teachers, as to how to ‘map and assess performance in the procedures of science’ (Donnelly *et al.*, 1996, p. 47). As well as there being no sound basis to inform the development of assessment, this was coupled with arguments that conducting whole science investigations was also ‘a major change in practice for most science teachers’ (Donnelly *et al.*, 1996, p. 8). This view was shared by a number of others (see e.g. Donnelly, 1994; Black, 1995a, 1995b; Jenkins, 1995b). Consequently, support texts and training from the examination boards and other organisations in how to conduct and assess Sc1 proliferated and became, as Jenkins (1995a) suggested, a ‘minor industry’ (p. 474). Therefore, as a result of the lack of firm theoretical and teaching capacity underpinnings, these texts were prescriptive and readerly. This readerly focus also made its way to the classroom in highly prescriptive writing frames and tick sheets used by students when doing Sc1 investigations. The proliferation of these texts and their prescriptive guidance was criticised by Donnelly (1994) because, while the responsibility for assessment was being put into teachers' hands, the level of prescription laid down actually undermined teachers' professionalism with teachers becoming passive recipients in decisions about assessment.

##### Narrowing of science practical experiences in schools

Over time, our analysis showed a number of effects on practical work with the introduction of Sc1. Firstly, Sc1 itself began to be perceived ‘primarily as an assessment exercise, not as an experience to learn scientific content, processes or attitudes’ (Nott & Wellington, 1999, p. 14). Secondly, teachers perceived that the introduction of Sc1, and also the NC as a whole, had decreased the amount and range of practical work in schools as they became hoop jumping assessment exercises (Donnelly, 1994; Nott & Wellington, 1999; Donnelly, 2000; Jenkins, 2000; Hulme & Coll, 2008). The reasons for this seem to be because to reach the higher levels of attainment only a few investigations were appropriate. Indeed, the report of the Science and Technology Committee of the House of Lords, Science Teaching in Schools, drawing on written evidence to the Committee from the Consortium of Local Education Authorities for the Provision of Science Services, concluded that this had led to ‘perhaps as few as 10 different investigations forming the bulk of science GCSE coursework throughout the country’ (House of Lords, 2006, p. 28).

##### Epistemological concerns about the Nature of Science investigations

Very early on in the introduction of Sc1 investigations concerns were raised about the way in which they were propagating unhelpful notions about the nature and philosophy of science (Tytler & Swatton, 1992; Donnelly, 1994; Jenkins, 1995a; Donnelly *et al*., 1996; Donnelly, 2001). Donnelly *et al. *(1996), capturing researchers' key concerns, particularly criticises the version of Sc1 that emerged in 1991, which focused on the control of variables, for promoting a version of the scientific method that was individualistic, out of date and simplistic.

Jenkins (2009), although acknowledging that the 1995 version had restored more accepted elements on the nature of science, maintained that Sc1 propagated a view of investigation in science, guided by variable control and fair testing, as ‘mechanistic’ and ‘routinised’ (p. 76). Tytler and Swatton (1992) also supported the view that Sc1, dominated by controlling variables, would ‘present a simplistic and ultimately misleading picture of how scientists conduct an investigation’ (p. 22). The responsibility for the reductive focus on variable control and fair testing was laid at the door of the Assessment Performance Unit (APU) a body set up in 1975 within the Department of Education and Science (DES) to promote the development of methods of assessing and monitoring the achievement of students at school (Donnelly, 1994). This, alongside the loss of AT17 (Nature of Science) from the 1989 science curriculum in the revised version of 1991, meant that more sophisticated and diverse understandings of how science works, more consistent with the philosophy of science and represented in AT17, were argued to have been replaced by these more simplistic notions located in empiricism and hypothesis testing.

##### Additional assessment challenges

As well as the foregoing assessment challenges, the assessment burden on teachers of marking Sc1 assessments continued to be reported in this period, since students often received feedback multiple times before the work was submitted for summative assessment (QCA, 2005, 2006a, 2006b; Ipsos MORI, 2006). Secondly, the high‐stakes nature of testing in England, coupled with the fact that Sc1 investigations were internally marked by teachers, led to reports of cheating on the part of teachers and schools and raised issues of reliability and authenticity in assessments. In particular, it had become unclear exactly who had completed coursework in many cases and there was evidence for the over‐involvement of parents (Bishop *et al*., 2006); the advent of the internet also introduced a plethora of support that also constituted cheating. Note that these issues were general to coursework and not specific to GCSE Science or practical science.

In summary, when writing about the implementation of Sc1 Millar (2011) commented:

Attainment target 1 (Scientific enquiry) has not been a success. It did not promote the kind of practical enquiry of those who first proposed it intended. Rather, it led to a routinisation of practical activity, and a reduction in the kind of illustrative practical work that can help students gain knowledge of natural phenomena and understanding of concepts and principles. (p. 180)

As a result of the challenges in the context of implementation reported here and a major revision of the NC, the assessment of practical work at GCSE changed to controlled assessment in the period 2006–2016.

### Era 2: 2006–2016—coursework assessment through controlled assessments

#### Context of influence

In many senses, the context of influence which shaped the decisions to introduce coursework by controlled assessment has already been outlined in detail in the previous section. In addition, a key curriculum influence on the revised version of the science NC implemented in 2006 was the *Beyond 2000* report (Millar & Osborne, 1998). This document presented an argument for a more significant place in the science curriculum for consideration of the nature of science or, as it is phrased in the 2006 NC document, *How Science Works.* The new orders for science maintained the structure of attainment targets and programmes of study but Sc1 at GCSE was now titled ‘How Science Works’ to reflect its focus on the nature of science, and this focus was much more closely aligned with the philosophy of science.

Investigative skills were, then, still a key component of the curriculum and assessment. However, development of practical skills in the curriculum was being given renewed emphasis in an increasingly hostile policy environment. Rising GCSE outcomes were contested, with competing explanations such as grade inflation (Coe, 2007) and cheating undermining their credibility with respect to validity and reliability. The coursework element of GCSE assessment underwent a significant reformation across all subjects, including science, to become what was termed ‘controlled assessment’. Addressing some of the concerns raised above the examinations regulator, Ofqual (2013), had the following aims for controlled assessments:

… to address concerns about the reliability and authenticity of coursework at GCSEs by improving the controls around setting tasks, the way work was produced by students and in the marking and moderation by teachers and exam board. (p. 3)

Controlled assessment of Sc1 now manifested itself differently, particularly in the core specifications, across the three examination boards and a summary of the assessment adopted by each examination board can be found in Table 3 below.

**Table 3:** Internal assessments in the 2006–2011 GCSE science specifications (adapted from SCORE, 2009)

	Specification	OCR twenty‐first century	OCR Gateway	AQA	Edexcel
Core	Total % internal assessment	33%	33%	25%	40%
	Assessment tasks	*20% Case study* (may not be practically based)	*Science in the News* (not practically based)	*ISAs* Written tasks based on a practical done by the student **PLUS** *Practical skills assessment*	*ISS* 3 written tasks based on practical work **PLUS** *Teacher‐assessed practical skills mark*
		*13.3% Data analysis task.* Based on primary data; collection of the data is not assessed	*Can‐do practical tasks* **OR** *Research task, Data analysis, Practical skills*		
Additional science	Total % internal assessment	33%	33%	25%	40%
	Assessment tasks	*A complete investigation*	*Research task, Data analysis, Practical skills*	As above	As above
Additional applied	Total % internal assessment	50%	n/a	60%	n/a
	Assessment tasks	*16.7% work related report* (not practically based) *21% practically based suitability test 12% standard procedures*	*Practical*	*20% Science in the workplace* (portfolio)	
				*40% Using Scientific skills* (portfolio)	

For example, the twenty‐first‐century Core Science assessment had two components: a case study and a data analysis activity. The case study involved students researching a science‐related question, giving them an opportunity to demonstrate their knowledge and understanding related to issues around the nature of science. Students studying the examination board OCR's Gateway Core Science could be assessed through practical tasks or a Science in the News task. The introduction of new modes of assessment in Sc1, such as the use of case study and tasks drawing on science in the news, have been advocated as authentic ways to assess the nature of science and science inquiry by some researchers (see e.g. Allchin, 2011). For the additional science qualification, taken in the second year of the GCSE, all boards maintained an investigative practical element.

#### Context of policy text production

The very name ‘controlled assessment’ implies a more readerly process of text production. Its readerly connotations are also supported in the guidance material produced by the three examination boards, as these demonstrate the level of control and guidance to be exercised by the examination boards on teachers showing little latitude for a more creative and writerly engagement of pupils and teachers with the controlled assessments. For example, the examination board AQA, in its guidance on controlled assessment, specifies in detail what happens in each of the three stages pupils have to undertake to ‘ensure reliability and authenticity’ (AQA, 2014, p. 4) and it also indicates the different levels of control teachers have to exercise in each of these three stages from limited control, with low level limited supervision, to high levels of control with formal supervision and work that must be undertaken effectively under examination conditions. Similar guidance can be found for the other two English examination boards, Edexcel and OCR.

#### Context of practice

Implementation of controlled assessment in science again attracted some concern,

Despite best efforts, since its introduction, controlled assessment has proved to be problematic in many ways, and some of those problems are intractable: it does not always assess those aspects of a subject it was put in place to assess, it can divert time from teaching and learning and be arduous to organise and deliver, and too often it is delivered inconsistently. (Ofqual, 2013, p. 2)

Teachers' workload (Ofqual, 2013) and variability in expertise to assess ‘How Science Works’ (SCORE, 2009)^3^ remained of concern. Additionally, variation between examination boards' approaches to the assessment was not always considered favourably (e.g. SCORE, 2009). Narrowing of the curriculum to the externally set assignments had resulted in prescriptive and repetitive teaching, and learning and assessment with questionable value for delivering the underlying practical science skills in pupils (Ofqual, 2015, p. 22).

Ironically, although controlled assessment was developed to address many of the concerns raised by Sc1, and indeed some of the examination boards used new and more authentic modes of assessment (case study and science in the news type tasks), the ensuing concerns about controlled assessment were remarkably similar. For example, controlled assessment also produced excessive workload associated with teacher assessment, was associated with lack of teacher expertise and, like Sc1, narrowed the range of practical work undertaken in schools. (OfQual, 2013, 2015) In addition, in our view it could also be argued that the level of control exercised through the guidance from examination boards in how to conduct controlled assessment, it could be argued, continued to contribute to the de‐professionalisation of science teachers.

### Era 3: 2016 to present day—written examination

#### Context of influence

A new version of the science NC was produced in 2016 returning explicitly to the three content areas of biology, chemistry and physics and retaining a strand related to practical science and how science works, called ‘working scientifically’.

The return to a more traditional formulation of biology, chemistry and physics was part of a broader policy drive by the then Minister of State for Education, Michael Gove, who, in 2010, raised concerns about the content and rigour of the science curriculum:

At the moment the science curriculum for those in Key Stage Three—preparing for GCSE—and for those pursuing Core and Additional Science at GCSE—doesn't even divide scientific knowledge into the discrete disciplines of physics, chemistry and biology. Instead there are hybrid headings—such as chemical and material behaviour or the environment, earth and universe—which take us further away from the essential disciplines of physics, chemistry and biology. (Gove, 2010)

In this period, the assessment of practical skills in the ‘working scientifically’ strand took a significantly different direction to the assessment of practical work through written examination. The introduction of a written examination is perhaps consistent with Gove's drive for higher standards (HC Deb, 2012). A significant impetus for the introduction of the written examinations can be located in an Ofqual consultation on how practical work in the new GCSEs should be assessed (Ofqual, 2015). This consultation drew on 172 responses from teachers and schools, awarding organisations, subject associations and learned societies, teacher representative groups, unions and employer and business groups.

The consultation sought views on a set of proposals as follows including: the introduction of written examinations which ‘would include questions that draw on students' practical science experience’, GCSE specifications laying down specific practicals and techniques where students keep their own records which would be available to exam boards on request (Ofqual, 2015, p. 8).

Embedded within these proposals are responses to issues and challenges identified in controlled assessment. For example, the setting of a written examination not marked by teachers was intended to reduce the assessment burden on teachers criticised in the controlled assessments. The report on the findings from the consultation showed broad support for the proposals above. Therefore, from September 2016 practical skills in the new GCSE are now assessed by written examination only and, as the consultation document stated, ‘students will now do practical work as part of their normal science lessons and, in the exam, there will be questions that will draw on these experiences of practical work’ (Ofqual, 2015, p. 8). The examinations will require students to demonstrate their understanding of scientific experimentation, with 15% of the total marks being allocated to each science GCSE (Ofqual, 2015). In the consultation document Ofqual argue that ‘this proportion is large enough to have a significant effect on a student's grade, but not so large as to distort assessment or hinder coverage of other requirements in these subjects’ (Ofqual, 2015, p. 8).

#### Context of policy text production

To support the decision to assess practical work by written examination in the ‘working scientifically’ strand, the three examination boards in England issued comprehensive guidance on the assessment of practical work in science at GCSE. For the examination boards, AQA and Edexcel, there are eight ‘required’ or ‘core’ practicals for each single GCSE in biology, chemistry and physics. OCR took a slightly different approach in each subject by identifying Practical Activity Groups (PAGs) and then giving examples of practicals that are suitable for the particular PAG. By doing these designated practicals, it is intended that students will acquire all the skills laid down by Ofqual which will then be tested in the written examinations. These developments, and the guidance provided by each examination board, on the one hand, suggest a more readerly set of policy guidelines for teachers to follow. AQA guidance, for example, has a ‘Required practical handbook’, which includes instructions for technicians, equipment lists, guidance for teachers and student method sheets with detailed instructions of how to carry out each practical task. The provision of these student method sheets, for example, arguably reduces practical work to the cook book recipe‐like format for students criticised in the past as a formulaic way of conducting practical work (see e.g. Clackson & Wright, 1992). On the other hand, it could be argued that the policy is also more writerly than that provided for the previous controlled assessments because teachers can now decide how and when to do these practicals, making more space for them to exercise their professional judgement. In addition, the consultation and guidance strongly suggest that schools will also be free to do other practical work in addition to the practical work suggested in each examination board's guidance, suggesting again the striking of a more writerly tone. However, only as the implementation of the new assessment unfolds will we be able to ascertain the ways and extent to which it has addressed the issues and challenges of the past.

## Discussion and conclusion

This paper has shown that the dual aims of developing and enacting a curriculum policy facilitating authentic and creative practices in practical work alongside a high‐stakes assessment policy at GCSE has been an intractable problem. Firstly, the fostering of authentic scientific inquiry is a complex task in itself, as Hume and Coll (2008) wrote:

Authentic scientific inquiry can thus be viewed as a complex social practice that involves participants interpreting, negotiating and justifying their inquiry approach in order to build believable and plausible explanations about how the physical world works. (p. 1203)

Secondly, the coupling high‐stakes assessment with fostering authentic scientific inquiry seems not to have been given sufficient policy or research attention, with previous authors often taking partial views of the issues. As we have shown in our analysis, the teaching and learning of investigative practical work for the GSCE became formulaic, narrow and dominated by hoop jumping exercises that bore little resemblance to the spirit of scientific inquiry or the nature of science intended by the SWP in the first two eras. The problems have not been stable and, as they evolved, led to unforeseen, negative, consequences elsewhere because they crossed the responsibilities of a number of organisations. In this case, initially two key ‘organisations’, the SWG and TGAT, were tasked with writing the science curriculum and the assessment of the curriculum as a whole, respectively. Our analysis suggests that both came to this task with innovative and creative ideas. There is evidence to suggest that the SWG led by Jeff Thompson wanted to embrace science investigations, inquiry and the nature of science as genuinely creative ways to teach science. Equally, Paul Black, in his leadership of TGAT, wanted to create an assessment system that broke away from the narrow summative assessments of the past and embrace diagnostic and formative assessment at a national level for the first time. However, the combination of the ambition of these two ‘organisations’ led to a number of unintended negative consequences. Practical inquiry and investigation work, rather than fostering creativity and diversity, became, as said, a narrow, reductive and formulaic exercise that bore little resemblance to its original intentions.

Manifold factors influenced these rather negative outcomes which we have reported on in detail above. However, the case of GCSE practical science in England is by no means unique in science education. Hume and Coll (2008) reported on the case of New Zealand and Year 11 students (15–16‐year olds) doing science investigations for Science Achievement Standard 1.1 Carrying out a practical investigation (SAS 1.1). They also reported on the intentions of a curriculum designed to foster creative and imaginative scientific inquiry, in a more writerly way, also being influenced, in unintended and negative ways, by high‐stakes assessment. The parallels to the English case are striking:

… students were acquiring a narrow view of scientific inquiry where thinking was characteristically rote and low‐level. The nature of this learning was strongly influenced by curriculum decisions made by classroom teachers and science departments in response to the assessment requirements of a high stakes national qualification. As a consequence of these decisions, students experienced structured teaching programmes in which they were exposed to content that limited the range of methods that scientists use to fair testing and to pedagogies that were substantially didactic in nature. (Hume & Coll, 2008, p. 1201)

They also reported the use of planning templates that resonates with a more readerly closed down translation of practical investigation which contributed to student learning that was ‘mechanistic and superficial rather than creative and critical’ (p. 1201).

Both contexts, England and New Zealand, began with policy aspirations to promote scientific investigation that was creative and authentic. In practice, this did not happen and, in both cases, a key factor which seems to have shaped policy enactment was practical investigations being inextricably linked to high‐stakes assessment.

The distorting effect of high‐stakes assessment therefore seems to be significant in both cases. Neither is the case of the distorting effects of high‐stakes assessment just a feature of assessment and curriculum policy in science in England and New Zealand. Berliner (2011) has shown in the US that high‐stakes assessment leads to curriculum narrowing with many resonances with the findings for the case in this article. Assessment is an important and integral part of learning, so the question is whether we can design assessments that can withstand the implications of high‐stakes testing.

If we know that the implications are likely to be teaching to the test, narrowing of the curriculum and gaming of the system, then we have to approach the assessment design with a much more nuanced view of what a ‘test worth teaching to’ would look like. This raises the question of whether assessment of practical science work can validly be assessed through coursework due to the twin problems of cheating and drilling, in addition to the above implications of high‐stakes testing. Although the idea of test worth teaching to has been in circulation for some time, it has tended to focus upon the design of authentic assessments or grade inflation, without criteria for deeming a test worth teaching to that tackles both of these issues (Koretz, 2005).

To discourage the narrow formulaic teaching described in this review, the assessment design should have broad curriculum coverage. A manageable curriculum volume is important to ensure that assessments can be designed in this way and teachers and learners can have a reasonable expectation of curriculum coverage. Engaging curriculum and assessment materials is important to provide disincentives to narrow curriculum coverage. Providing teachers with a framework so that they can understand the rationale for the assessment design is important so that they can engage professionally with the curriculum and assessment materials. This signals expectations and supports a professional approach to pedagogy. As part of this, progression models, showing what it means to have learned the material are important, so that teachers do not have to construct learning theories for themselves in relation to the curriculum and assessment materials. Crediting higher order thinking skills (as appropriate) in the scoring rubrics is also important in attempting to thwart superficial coaching and drilling, though this is not failsafe in itself. Finally, mechanisms for verifying the source and conditions of the performances are important to ensure that the validity of the scores is not undermined by cheating.

For these reasons, recent reforms at A level (national examinations taken at age 18 in England) have an endorsement by the teacher to indicate that practical work has been conducted, rather than an assessment whose marks are included in the final grade. Early findings imply that this has not led to the feared reduction in practical skills of undergraduates (Cadwallader, 2019). As such, taking the assessment at a remove from the high‐stakes environment may be a different solution. Alternatively, designing the assessment in such a way that narrowing of the curriculum and drilling become less feasible or desirable may be possible. There are lessons here regarding assessment policy making. Designing the curriculum and assessments separately, or bemoaning the effects of one upon the other fails to take into account what we have learned over the past 30 years regarding their relationship. The labour market has changed over this timeframe too, with a paucity of opportunities for a good life for those without competitive qualifications and skills. Evidence‐based design needs to build upon concrete cases such as what we know about the design and implementation of practical science assessment policy.

## Conflict of interest

The authors report no conflict of interest.

## Geolocation information

There is no dataset associated with this submission; therefore, data deposition and geolocation information are not applicable.

## Notes

1 General Certificate of Secondary Education (GCSE), normally taken at age 16 and Advanced levels (A levels), normally taken at age 18.

2 Sc1 is the name given to practical assessment at GCSE (see discussion later).

3 Science Community Representing Education.
